# Association between insomnia and subclinical atherosclerosis among Chinese steelworkers: a cross-sectional survey

**DOI:** 10.1186/s13690-022-00834-1

**Published:** 2022-03-14

**Authors:** Lihua Wang, Shengkui Zhang, Miao Yu, Juxiang Yuan

**Affiliations:** Department of Epidemiology and Health Statistics, School of Public Health. North China University of Science and Technology, Tangshan, Hebei Province China

**Keywords:** Insomnia, Carotid plaque, Subclinical atherosclerosis, Steelworker

## Abstract

**Background:**

Insomnia is a common prevalent sleep disorder. Difficulty maintaining sleep or poor in quality in insomnia caused by disrupted or misaligned circadian rhythms may play an important role in the development of atherosclerosis. This study aimed to examine the association between insomnia and subclinical atherosclerosis in Chinese steelworkers.

**Methods:**

A total of 3240 subjects from a large enterprise located in northern China were included in this study. The Athens Insomnia Scale (AIS) was used to assess the status of insomnia. Subclinical atherosclerosis was evaluated using ultrasonographic measurements of carotid plaque. Multivariable logistic regression was used to identify association between insomnia and carotid atherosclerosis.

**Results:**

The overall prevalence of insomnia and carotid plaque were 35.3 and 31.7% in the study population. Compared with non-insomnia workers, significantly increased odds of carotid plaque were observed among insomnia workers after adjusting for potential confounders, odds ratio (OR) = 1.38, 95% confidence interval (CI): 1.17 to 1.63. Exposure to current shift work and insomnia simultaneously can significantly elevated the odds of carotid plaque.

**Conclusion:**

Insomnia is associated with elevated odds of carotid atherosclerosis in male steelworkers. Insomnia problems of workers should receive further attention in occupational worker health interventions.

**Supplementary Information:**

The online version contains supplementary material available at 10.1186/s13690-022-00834-1.

## Background

Atherosclerotic cardiovascular disease (ASCVD) nowadays accounts for the majority of mortality worldwide [1]. Atherosclerosis is a chronic arterial disease and a major cause of vascular death [2]. The pathophysiological mechanisms of atherosclerosis mainly involves lipid and lipoprotein metabolisms, lipid accumulation in macrophages, inflammation and immune cell lipid loading, endothelial dysfunction, smooth muscle cells proliferation, apoptosis and senescence, and comprehensive regulation of small noncoding RNAs [3, 4]. However, cardiovascular disease (CVD) develops over a long period of time with physical changes beginning decades before the disease manifests itself. Therefore, it is important to include surrogate parameters that describe early subclinical changes [5]. The measure of subclinical atherosclerosis in several body locations, such as carotid, femoral and coronary arteries, allows one to find evidence of vascular disease before it causes symptoms [6].

Insomnia is a common prevalent sleep disorder characterized by difficulty initiating or maintaining sleep, accompanied by symptoms such as irritability or fatigue during wakefulness [7]. The prevalence of insomnia varies from 6 to 48% in more than 50 epidemiological studies according to different definitions [8], suggesting that individuals with insomnia constitute a considerable proportion and insomnia has become one of the most common and widely recognized public health problems on a global scale [9]. Insomnia is a risk factor for the emergence and development of numerous somatic and mental disorders [10] and follows a chronic course in 40-70% of individuals over 1-20 years [7]. Functional consequences of insomnia including reduced productivity, increased absenteeism, and increased health care costs and a declined quality of working life [11–13].

A substantial percentage of shift workers often experience reduced sleep quality, duration and/or excessive sleepiness due to the imposed conflict between work and their circadian system [14]. It is estimated that about 32% of night workers, 10% of day workers [15], and 8-26% of rotating shift workers [16, 17] suffer from shift work disorder (SWD), a circadian rhythm sleep disorder characterized by insomnia and/or excessive sleepiness as a result of chronic mismatch between shift workers’ sleep-wake schedule and circadian clock [18]. Disruption or maladjustment of circadian rhythms causes varying degrees and types of sleep problems, and ultimately leads to the development of atherosclerosis through its effects on the autonomic nervous system and chronic inflammation [19, 20]. However, the results of several studies on insomnia and subclinical atherosclerosis were inconsistent [19, 21] and data from population-based studies sparse. The aim of our study was to examine the relation between insomnia and carotid plaque in the steelworkers.

## Methods

### Study design and population

This study was based on cross-sectional data from the occupational population, which was conducted among steelworkers at 11 steel production departments owned by the HBIS Group’s Tangsteel Company in Tangshan City, Hebei Province in North China. All workers at this company underwent a legally required health examination each year. A total of 7661 participants who underwent the required annual legally occupational health examinations were recruited from February to June 2017. There were 4084 workers who volunteered and completed carotid ultrasound examinations. After excluding 97 workers without complete items in the insomnia scale, 205 workers without sufficient shift work data, 200 workers without complete information on main covariates on the questionnaire, 342 female workers which less than 10%, a total of 3240 participants were included in the final analysis. All participants gave informed consent before taking part in this study. The research was approved by the Ethics Committee of North China University of Science and Technology (No.16040).

### Assessment of insomnia

The entire 8-item Athens Insomnia Scale (AIS) based on the International Classification of Diseases, Tenth Revision (ICD-10) diagnostic criteria of insomnia was employed as the insomnia assessment tool in this study [22]. The first 5 items evaluate difficulty with sleep induction, awakening during the night, early-morning awakening, total sleep time, and overall quality of sleep; the last 3 items focus on sense of well-being, overall functioning, and sleepiness during daytime. Each item was scored on a 4-point Likert scale from 0 (no problem at all) to 3 (a very serious problem), with the total score ranging from 0 to 24. A score of ≥6, which being the widely accepted cut-off value for insomnia, classified the workers into the insomnia group; other workers were classified as the non-insomnia group.

### Measurement of plaque in the carotid artery

The assessment of plaque from both the left and right carotid artery systems was performed by two trained sonographers who used a high-resolution B-mode topographic ultrasound system (PHILIPS, HD7, China) and were blinded to the research purpose and the study design. Participants were examined in the supine position with their head rotated in the opposite direction to the probe and with a lateral probe orientation. For this study, atherosclerotic plaques were defined as focal structures encroaching into the arterial lumen of at least 0.5 mm or 50% of the surrounding IMT value, or demonstrating a thickness > 1.5 mm as measured from the intima-lumen interface to the media-adventitia interface [23]. When a local protrusion was defined as a plaque, its maximum thickness (mm) was measured using ultrasound calipers [24]. The carotid plaque score, which indicates the severity of atherosclerosis, is the sum of the cumulative maximum thickness of plaques obtained in the longitudinal sections of the common carotid artery, bifurcation, and internal carotid artery of the left and right carotid systems [25].

### Assessment of covariates

Information on demographic characteristics, work lifestyle behavior, clinical characteristics were collected via face-to-face questionnaire survey, physical and biochemical examination. The duration of sleep was the weighted averages of sleep on working days and rest days, and divided into two groups according to 6 h. Habitual snoring was defined as a self-report of snoring >4 times per week. Workers were asked if they had taken any sleeping pills in the past month. Smoking status was divided into “never”, “ever” and “current”. Those who regularly consumed ≥1 cigarette/day over the past 12 months were defined as current smokers. Drinking status was divided into “never”, “ever” and “current”. Those who usually consumed some alcohol at least once a week over the past 12 months were defined as current drinkers. Considering that current lifestyle habits have a more significant effect on insomnia, and the proportion of past smokers, past drinkers and former shift workers were smaller, we divided these three variables into two categories according to “current” state. Dietary patterns were assessed based on the DASH (dietary approaches to stop hypertension, DASH) diet score [26]. The calculation of metabolic equivalents was based on the International Physical Activity Questionnaire (IPAQ) [27]. The workers with metabolic equivalent task (MET) [min/week] values <600, 600-3000 and > 3000 were classified as having a low, moderate, and high level of physical activity respectively [28]. Body mass index (BMI) was defined as body weight (kg) divided by the square of the body height (m^2^). The main work schedule of the present study population has been introduced in detail in our previous research [29]. In brief, shift work refers to rotating night shifts (working through 00:00 to 6:00; the mainly four−crew−three−shift system now and historical three−crew−two−shift system) and was divided into never/ever and current according to the current shift status. Hypertension was defined as current systolic blood pressure ≥ 140 mmHg, or diastolic blood pressure ≥ 90 mmHg, or if the patient was receiving antihypertensive therapy [30]. Diabetes was defined as fasting blood glucose ≥7.0 mmol/L or if the patient was receiving hypoglycemic therapy [31]. Total cholesterol (TC) ≥ 6.2 mmol/L or low-density lipoprotein (LDL-C) ≥ 4.1 mmol/L or high-density lipoprotein (HDL-C) < 1.0 mmol/L or triglycerides (TG) ≥ 2.3 mmol/L, or patients undergoing lipid-lowering therapy were considered to demonstrate dyslipidemia [32]. Four mainly related occupational hazard factors (heat stress, noise, dust and carbon monoxide) were measured by a qualified third-party company in accordance with the National Occupational Health Standards of the People’s Republic of China (see Supplementary file 1).

### Statistical analysis

Continuous variables are presented as means and standard deviations, and between-group comparisons were performed using Student’s t-test if the data were normally distributed. Otherwise, the median (upper quartile-lower quartile) and Wilcoxon rank sum test were used to describe and compare the continuous variables between the groups. Categorical variables are presented as numbers and percentages, and the chi-square test was used to compare differences among groups. Associations between insomnia and carotid plaque were reported as odds ratios (ORs) and the corresponding 95% confidence intervals from multivariable adjusted logistic regression models. The risk factors and potential confounders were included in the analysis. We fit an unadjusted model and a fully adjusted model which including potential confounders and known risk factors as follows: age, educational level, BMI (categorical), smoking status, drinking status, shift work, sleep duration (categorical), sleep drug, snore, hypertension, diabetes and dyslipidemia. Subsequently, in subgroup analysis, we introduced multiplicative interaction terms using the insomnia and the stratifying factors including BMI (< 25 kg/m^2^ or ≥ 25 kg/m^2^), smoking status (no/current), drinking status (no/current), snore (no/yes), shift work (no/current), sleep duration (< 6 h or ≥ 6 h), diabetes (no/yes), hypertension (no/yes), and dyslipidemia (no/yes) to assess potential effect modification. The log likelihood ratio test was used to compare models with and without cross-product interaction terms. A two-tailed *p* < 0.05 was considered statistically significant. All statistical analyses were performed using SAS V.9.4 (SAS Institute, Cary, NC, USA).

## Results

### General characteristics of the participants

Table 1 shows the general characteristics of the study participants according to insomnia status. The present study of 3240 included participants with a mean age of 46.1 years. Among the included workers, the prevalence of insomnia was 35.3%, and the proportion of current shift workers was 60.1%. Presence of plaque, diabetes, snore, sleep drug and current shift were more likely to be reported among insomnia workers. Compared with non-insomnia workers, the sleep duration was relatively shorter and the DASH score was relatively low among insomnia workers. In terms of current health status, insomnia workers also showed higher levels of systolic blood pressure and diastolic blood pressure. Workers with carotid plaque were more likely to be current shift workers, current smokers, current drinkers, and those with hypertension, diabetes or dyslipidemia (Table S1).

**Table 1:** Basic characteristics of participants according to insomnia. China, 2017

Variables	Total	Non-insomnia	Insomnia	***p***-Value
	***N*** = 3240	***n*** = 2097	***n*** = 1143	
Plaque, n (%)	1028 (31.7)	644 (29.7)	426 (35.5)	<0.001
Plaque score (mm), median (IQR)	2.8 (1.8, 4.8)	3.2 (1.9, 5.3)	2.3 (1.6, 4.0)	<0.001
Age (years), mean (SD)	46.1 (8.1)	46.1 (8.2)	46.2 (7.9)	0.627
Sleep duration (h), mean (SD)	6.8 (1.2)	6.8 (1.1)	6.6 (1.3)	<0.001
DASH score, mean (SD)	21.4 (2.4)	21.5 (2.4)	21.3 (2.3)	0.014
Physical activity (MET-h/week), median (IQR)	113.0 (79.6, 158.7)	111.3 (77.6, 158.3)	121.6 (83.3, 167.8)	<0.001
BMI (kg/m^2^), mean (SD)	25.3 (3.3)	25.4 (3.3)	25.3 (3.3)	0.530
Systolic blood pressure (mmHg), mean (SD)	130.3 (16.6)	129.9 (16.3)	131.2 (17.0)	0.031
Diastolic blood pressure (mmHg), mean (SD)	83.3 (10.6)	83.1 (10.4)	83.8 (10.9)	0.078
Fasting blood glucose (mmol/L), mean (SD)	6.2 (1.4)	6.1 (1.4)	6.2 (1.4)	0.510
Total cholesterol (mmol/L), mean (SD)	5.1 (1.0)	5.1 (1.0)	5.2 (1.0)	0.271
Triglycerides (mmol/L), mean (SD)	1.7 (1.6)	1.7 (1.6)	1.8 (1.6)	0.753
HDL-C (mmol/L), mean (SD)	1.3 (0.3)	1.3 (0.3)	1.3 (0.3)	0.155
LDL-C (mmol/L), mean (SD)	3.3 (0.9)	3.2 (0.9)	3.3 (0.9)	0.183
**Age (years), n (%)**				0.398
23–29	155 (4.8)	106 (5.1)	49 (4.3)	
30–39	532 (16.4)	349 (16.6)	183 (16.0)	
40–49	1234 (38.1)	778 (37.1)	456 (39.9)	
50–60	1319 (40.7)	864 (41.2)	455 (39.8)	
**Education level, n (%)**				0.994
Primary or Middle	969 (29.9)	627 (29.9)	342 (29.9)	
High school or college	1707 (52.7)	1106 (52.7)	601 (52.6)	
University and above	564 (17.4)	364 (17.4)	200 (17.5)	
**Marital status, n (%)**				0.630
Unmarried	102 (3.2)	69 (3.3)	33 (2.9)	
Married	3059 (94.4)	1980 (94.4)	1079 (94.4)	
Other	79 (2.4)	48 (2.3)	31 (2.7)	
**Smoking status, n (%)**				0.820
Never/Ever	1409 (43.5)	915 (43.6)	494 (43.2)	
Current	1831 (56.5)	1182 (56.4)	649 (56.8)	
**Drinking status, n (%)**				0.122
Never/Ever	1890 (58.3)	1244 (59.3)	646 (56.5)	
Current	1350 (41.7)	853 (40.7)	497 (43.5)	
**DASH score, n (%)**				0.024
<20	573 (17.7)	346 (16.5)	227 (19.9)	
20–21	1083 (33.4)	688 (32.8)	395 (34.6)	
22–23	1034 (31.9)	691 (33.0)	343 (30.0)	
≥24	550 (17.0)	372 (17.7)	178 (15.6)	
**Physical activity, n (%)**				0.290
Low	35 (1.1)	27 (1.3)	8 (0.7)	
Moderate	226 (7.0)	148 (7.1)	78 (6.8)	
High	2979 (91.9)	1922 (91.6)	1057 (92.5)	
**BMI (kg/m**^**2**^**), n (%)**				0.762
<25	1559 (48.1)	1000 (47.7)	559 (48.9)	
25–30	1417 (43.7)	927 (44.2)	490 (42.9)	
≥30	264 (8.2)	170 (8.1)	94 (8.2)	
**Shift work, n (%)**				0.021
Never/Ever	1292 (39.9)	867 (41.3)	425 (37.2)	
Current	1948 (60.1)	1230 (58.7)	718 (62.8)	
**Sleep duration (hour), n (%)**				<0.001
<6	399 (12.3)	187 (8.9)	212 (18.6)	
≥6	2841 (87.7)	1910 (91.1)	931 (81.4)	
**Snore, n (%)**	956 (29.5)	575 (27.4)	381 (33.3)	<0.001
**Sleep drug, n (%)**	151 (4.7)	68 (3.2)	83 (7.3)	<0.001
**Hypertension, n (%)**	1093 (33.7)	692 (33.0)	401 (35.1)	0.231
**Diabetes, n (%)**	465 (14.4)	283 (13.5)	182 (15.9)	0.060
**Dyslipidemia, n (%)**	1321 (40.8)	866 (41.3)	455 (39.8)	0.410

Values are expressed as the mean (SD) or median (IQR) or number (%); *p* values were from Pearson’s χ ^2^ test for categorical variables and Student’s t-test or Wilcoxon rank sum test for continuous variables. *DASH* dietary approaches to stop hypertension, *MET* metabolic equivalent of task, *BMI* body mass index, *HDL-C* high density lipoprotein cholesterol, *LDL-C* low density lipoprotein cholesterol

### Association between insomnia and carotid plaque

The overall prevalence of carotid plaque was 31.7% in this study population (Table 1). Table 2 shows the results from the logistic regression model. Compared with non-insomnia workers, significantly increased the odds ratios (ORs) of carotid plaque were observed in insomnia workers in unadjusted model (OR = 1.31, 95% CI: 1.12 to 1.52). After additionally adjusting for age, educational level, BMI, smoking status, drinking status, shift work, sleep duration, snore, sleep drug, diabetes, hypertension and dyslipidemia, this association remained robust (OR = 1.38, 95% CI: 1.17 to 1.63) (Table 2). In the cross-classification analyses, compared with workers who never/ever shift work, non-insomnia and sleep duration ≥6 h, those who exposure to current shift work and insomnia simultaneously showed significantly elevated odds of carotid plaque (OR = 2.30, 95% CI: 1.78 to 2.96, in sleep duration ≥6 h group; OR = 1.64, 95% CI: 1.10 to 2.46, in sleep duration <6 h group), (Table 3). However, no significant additive interactions between the shift work and insomnia on the odds of carotid plaque were observed (Table S2 and Fig. S1). We analyzed the relationship between insomnia and carotid plaque through stratification analysis based on potential effect modifiers (Table 4). Compared with non-insomnia workers, elevated odds of carotid plaque were observed in insomnia workers in almost all subgroup analyses. There was no significant effect modification of the association between insomnia and carotid plaque by smoking status, drinking status, BMI, DASH score, hypertension, diabetes and dyslipidemia (all *p* for interaction >0.05). Considering that dust, heat stress, noise, and carbon monoxide are the main occupational hazards for current steelworkers, we further adjusted these exposures on the basis of the fully adjusted model in Table 2, and the results remained robust (Table S3).

**Table 2:** The Odds Ratio (OR) of carotid plaque in steelworkers with insomnia compare to non-insomnia. Multivariate logistic regression China, 2017

Insomnia	Total, n (%)	OR (95% CI)	
		Unadjusted	Adjusted
No	2097 (64.7)	1.00	1.00
Yes	1143 (35.3)	1.31 (1.12 to 1.52)	1.38 (1.17 to 1.64)

Adjusted for age, educational level, BMI (categorical), smoking status, drinking status, shift work, sleep duration (categorical), sleep drug, snore, hypertension, diabetes and dyslipidemia

**Table 3:** Association between insomnia, shift work and carotid plaque Odds Ratio (OR). Cross-classification analysis China, 2017

Shift work	Insomnia	Sleep duration	n (%)	OR (95% CI)
Never/Ever	No	≥6 h	806 (24.9)	1.00
Never/Ever	No	<6 h	61 (1.9)	1.31 (0.73 to 2.36)
Never/Ever	Yes	≥6 h	358 (11.0)	1.35 (0.97 to 1.84)
Never/Ever	Yes	<6 h	67 (2.1)	1.76 (0.99 to 3.27)
Current	No	≥6 h	1104 (34.1)	1.62 (1.01 to 2.10)
Current	No	<6 h	126 (3.9)	1.17 (0.76 to 1.81)
Current	Yes	≥6 h	573 (17.7)	2.30 (1.78 to 2.96)
Current	Yes	<6 h	145 (4.5)	1.64 (1.10 to 2.46)

Adjusted for age, educational level, BMI (categorical), smoking status, drinking status, shift work, sleep duration (categorical), sleep drug, snore, hypertension, diabetes, dyslipidemia

**Table 4:** The Odds Ratio (OR) of carotid plaque in steelworkers with insomnia compare to non-insomnia. Stratification analysis by smoking status, drinking status, BMI, DASH score, hypertension, diabetes and dyslipidemia China, 2017

Characteristics	OR (95% CI)		***p***_**Interaction**_
	Non-insomnia	Insomnia	
**Smoking status**			0.759
Never/Ever	1.00	1.43 (1.10 to 1.87)	
Current	1.00	1.35 (1.09 to 1.68)	
**Drinking status**			0.051
Never/Ever	1.00	1.65 (1.31 to 2.07)	
Current	1.00	1.10 (0.85 to 1.43)	
**BMI (kg/m**^**2**^**)**			0.510
<25	1.00	1.47 (1.15 to 1.89)	
≥25	1.00	1.32 (1.04 to 1.66)	
**DASH score**			0.378
<21	1.00	1.25 (0.98 to 1.60)	
≥22	1.00	1.51 (1.19 to 1.92)	
**Hypertension**			0.339
No	1.00	1.25 (1.01 to 1.56)	
Yes	1.00	1.61 (1.22 to 2.12)	
**Diabetes**			0.459
No	1.00	1.42 (1.18 to 1.72)	
Yes	1.00	1.21 (0.80 to 1.83)	
**Dyslipidemia**			0.147
No	1.00	1.61 (1.28 to 2.02)	
Yes	1.00	1.12 (0.86 to 1.45)	

*p-*values for interaction were estimated using a log likelihood ratio test to compare models with and without cross-product interaction terms. *p* Interaction were derived by insomnia × characteristics in Models that adjusted for age, educational level, BMI (categorical), smoking status, drinking status, shift work, sleep duration (categorical), sleep drug, snore, hypertension, diabetes, dyslipidemia (except for the stratification variable in each subgroup)

## Discussion

In this cross-sectional study of occupational populations, we examined the association between insomnia and subclinical atherosclerosis. The prevalence of carotid plaque among insomnia workers and non-insomnia workers were 35.5 and 29.7%, respectively. Positive associations were observed between insomnia and the odds of carotid plaque after adjusting for possible confounding factors, adding evidence to an underlying pro-atherogenic role of insomnia in ASCVD.

An ultrasound examination of the carotid arteries is an easily accessible tool by which one can identify early subclinical atherosclerosis through the detection of plaques. In concrete, the presence of carotid plaques has been shown to significantly improve the better [33] risk prediction of major cardiovascular events [34–36], and the accuracy of diagnosing of coronary artery disease [37]. Previous study found that carotid intima media thickness (CIMT) was significantly greater in insomnia group and insomnia with short sleep duration was associated with high plaque score [19]. Another study among midlife women found that shorter objective sleep time and poorer subjective sleep quality were associated with higher mean IMT and higher odds of carotid plaque adjusting for CVD risk factors, hot flashes, and estradiol [38]. Carotid plaque is a more direct measure of atherosclerotic lesion development, and has been shown to significantly improve the risk prediction of CVD risk factors than CIMT [38, 39]. Our findings extend the positive association between insomnia and the odds of carotid plaque by adding findings that when insomnia, the sleep duration and shift work are considered simultaneously, significantly higher odds of carotid plaque are limited to participants who have been exposed to shift work and insomnia. Shift work cannot be completely avoided in industrial production. This implies that improving insomnia symptoms, or transferring workers with severe insomnia from shift work to day work, may reduce the odds of carotid plaque. Our findings contribute to the discussion on the importance of good sleep status and shift work and carotid plaque, and may have important public health implications, mainly in relation to primary prevention. When preparing the appropriate strategies for the prevention and treatment of insomnia and/or ASCVD among steel workers, policy-makers are recommended to pay more attention to occupational factors. Health interventions and preventive services are needed for related workers to promote ASCVD health risk appraisal, adopt healthy lifestyle, improve their health status and reduce workplace costs.

The exact mechanisms by which sleep disorders may increase risk for atherosclerosis, stroke and vascular disease have not well been established. Several probable pathways are likely to underlie the association between insomnia and subclinical atherosclerosis. One potential mechanism is the presence of psychological and psychosocial stressors [40]. Shift workers are subjected to heavier stress loads (such as job strain or community-wide events) [41], and suffer mental health problems (such as depression or fatigue) than non-shift workers [42]. One of the principal mechanisms translating chronic stress into adverse cardio-metabolic outcomes is the up-regulation of the hypothalamic pituitary adrenal (HPA) axis [43]. In addition, the consequences of sleep deprivation to the sympathetic system and endocrine system have been demonstrated both in animals and insomnia subjects by increasing sympathetic activity, causing vascular endothelial dysfunction [44], elevating evening corticotropin and cortisol levels [45], reducing natural killer cell activity and increasing pro-inflammatory cytokines [46]. These could be the important underlying pathophysiological mechanisms of atherosclerosis. Furthermore, unhealthy behavioral lifestyle behaviors (e.g., less exercise, alcohol consumption) can increase the risk of insomnia [47]. Insomnia is also associated with higher nighttime systolic blood pressure, blunted dipping of nocturnal blood pressure [48], higher risks of developing hypertension [49], diabetes [50, 51] and dyslipidemia [52]. In our study, insomnia workers showed shorter sleep duration, less physical activity and higher levels of systolic blood pressure and diastolic blood pressure than non-insomnia workers. All these are correlated with increased risk of CVD and risk factors, have the potential to rupture vulnerable plaque and facilitate thrombus formation, resulting in myocardial infarction or sudden death [53].

The major strengths of our study include a large sample size and accurate calculation of carotid plaque by ultrasonography. However, our research also has certain limitations. First, we were unable to draw any causal inferences between insomnia and carotid plaque according to a cross-sectional study. Second, even though AIS is a widely used epidemiological tool to assess insomnia, it is not used for clinical diagnosis of insomnia. we were not able to obtain objective measures of sleep by polysomnography, the use of self-reported snoring more than 4 times a week as a surrogate for sleep disordered breathing may dilute any effects on carotid plaque by including subjects with primary snoring (no apneas) or mild sleep disordered breathing. Third, our study did not collect information about physical pain and mental disorders, which may have an impact on insomnia, although we did take sleep drug and snore into account. Possible recall and report errors and potential influencing factors in the above questions are the main information bias and residual confounding in this study. Fourth, our survey participants are currently participating in the standard four-crew-three-shift system, and other different shift systems were only found during the historical period, which made it impossible to compare between insomnia and different types of shift systems. However, it can be postulated that shift workers experience insomnia at each sleeping period related to their work schedule [54]. Fifth, long-term shift workers tend to have better physical condition (the healthy worker effect), which makes the association between exposure and outcome easy to underestimate. Finally, as our study was conducted in a steel production occupational setting where more than 90% steelworkers are male workers, generalization to other populations remains limited.

## Conclusion

In conclusion, insomnia is association with subclinical atherosclerosis in male steelworkers. Insomnia problems of workers should receive further attention in occupational worker health interventions. Further large-scale prospective longitudinal studies are warranted to confirm our findings.

## Supplementary Information

**Additional file 1: Table S1.** Basic characteristics of participants according to current carotid plaque status. China, 2017. **Table S2.** Additive interactions between shift work and insomnia on odds of carotid plaque. China, 2017. **Figure S1.** Relative risk with contributions from different exposure categories marked in additive interaction. U is the common reference category. **Table S3.** The Odds Ratio (OR) of carotid plaque in steelworkers with insomnia compare to non-insomnia. Multivariate logistic regression. China, 2017.

## Data Availability

The datasets generated and analyzed in the course of this study are available from the corresponding author on reasonable request.

## Abbreviations

AIS: Athens Insomnia Scale
DASH: Dietary approaches to stop hypertension
MET: Metabolic equivalent of task
BMI: Body mass index
HDL-C: High density lipoprotein cholesterol
LDL-C: Low density lipoprotein cholesterol
OR: Odds ratio
CI: Confidence interval
ASCVD: Atherosclerotic cardiovascular disease
CVD: Cardiovascular diseases
SWD: Shift work disorder
