# Supplementary material for: Association between insomnia and subclinical atherosclerosis among Chinese steelworkers: a cross-sectional survey
Source: Arch Public Health. 2022 Mar 14;80:80. doi: 10.1186/s13690-022-00834-1 (PMC8919587; doi:10.1186/s13690-022-00834-1)
Supplement: Supplementary file 1 — Additional file 1: Table S1. Basic characteristics of participants according to current carotid plaque status. China, 2017. Table S2. Additive interactions between shift work and insomnia on odds of carotid plaque. China, 2017. Figure S1. Relative risk with contributions from different exposure categories marked in additive interaction. U is the common reference category. Table S3. The Odds Ratio (OR) of carotid plaque in steelworkers with insomnia compare to non-insomnia. Multivariate logistic regression. China, 2017. [file 13690_2022_834_MOESM1_ESM.docx]

**Supplementary file**

**Association between insomnia and subclinical atherosclerosis among Chinese** **steelworkers: a cross-sectional survey**

**Assessment of main occupational hazards**

Exposure to dust was defined as workers who may be exposed to productive dust (inorganic dust, organic dust or mixed dust) during production (GBZ/T 229.1–2010). The total dust in the air of workplace was collected at the breathing zone with a filter membrane, and it’s concentration was calculated based on the increased weight of the filter membrane and the amount of gas collected. When the dust concentration in the air ≤50 mg/m^3^, a filter membrane with a diameter of 37mm or 40mm was used, otherwise a filter membrane with a diameter of 75mm would be used (GBZ/T 192.1–2007).[1]

Exposure to high temperature (heat stress work) was defined as the average wet-bulb globe temperature (WBGT) index of the workplace is equal or greater than 25℃ in the process of production (GBZ 2.2–2007).[2] The WBGT index was measured by black-wet bulb globe thermometer. If there was no productive heat source in the workplace, three measuring points were selected to take the average value of WBGT index, while where there was a productive heat source, 3 to 5 measuring points were selected to take the average value of WBGT index. If the workplace was isolated into different thermal or ventilated environment, 2 measuring points were selected to take the average value of WBGT index (GBZ/T 189.7–2007).[3]

Exposure to industrial toxicant was defined as workers who may be exposed to a variety of harmful chemicals (the toxicant specifically refers to carbon monoxide in this population) during production (GBZ/T 229.2–2010).[4] Carbon monoxide or carbon dioxide in the air of workplace was pumped into the Non-Dispersive Infrared-Ray (NDIR) analyzer and selectively absorbs their infrared rays. The concentration of carbon monoxide was determined according to the absorption value (GBZ/T 160.28–2004).[5]

Exposure to noise was defined as workers who exposed to a noisy environment where the 8h/d or 40h/week equivalent A-weighted sound pressure level is ≥80dB, which may be harmful to health and hearing (GBZ/T 229.4–2012).[6] The workplace production noise was measured by a sound level meter. If the distribution of sound field in the workplace was uniform (between-field difference of A-sound levels were less than 3dB(A)), three measuring points were selected to take the average value, otherwise it should be divided into several sound level areas. In each sound field, two measuring points were selected to take the average value (GBZ/T 189.8–2007).[7]

**Table of contents**

**Table S1** Basic characteristics of participants according to current carotid plaque status. China, 2017

| **Variables** | **Total** | **Without plaque** | **With plaque** | ***p*-Value** |
| --- | --- | --- | --- | --- |
|  | **N = 3240** | **n = 2212** | **n = 1028** |  |
| Insomnia, n (%) | 1143 (35.3) | 737 (33.3) | 406 (39.5) | <0.001 |
| Age (years), mean (SD) | 46.13 (8.10) | 44.32 (8.35) | 50.01 (5.90) | <0.001 |
| Sleep duration (h), mean (SD) | 6.75 (1.20) | 6.80 (1.19) | 6.66 (1.22) | 0.002 |
| DASH score, mean (SD) | 21.45 (2.35) | 21.40 (2.29) | 21.55 (2.49) | 0.104 |
| Physical activity (MET-h/week), median (IQR) | 113.0 (79.6, 158.7) | 117.0 (79.6, 159.6) | 114.1 (79.6, 157.6) | 0.335 |
| BMI (kg/m^2^), mean (SD) | 25.34 (3.29) | 25.28 (3.32) | 25.49 (3.23) | 0.097 |
| Systolic blood pressure (mmHg), mean (SD) | 130.3 (16.6) | 127.9 (15.7) | 135.5 (17.3) | <0.001 |
| Diastolic blood pressure (mmHg), mean (SD) | 83.3 (10.6) | 82.1 (10.3) | 86.0 (10.8) | <0.001 |
| Fasting blood glucose (mmol/L), mean (SD) | 6.2 (1.4) | 6.0 (1.2) | 6.4 (1.7) | <0.001 |
| Total cholesterol (mmol/L), mean (SD) | 5.15 (0.98) | 5.03 (0.94) | 5.42 (1.02) | <0.001 |
| Triglycerides (mmol/L), mean (SD) | 1.74 (1.59) | 1.75 (1.66) | 1.74 (1.43) | 0.899 |
| HDL-C (mmol/L), mean (SD) | 1.29 (0.32) | 1.27 (0.31) | 1.32 (0.34) | <0.001 |
| LDL-C (mmol/L), mean (SD) | 3.26 (0.87) | 3.16 (0.83) | 3.49 (0.91) | <0.001 |
| Age (years), n (%) |  |  |  | <0.001 |
| 23–29 | 155 (4.8) | 153 (6.9) | 2 (0.2) |  |
| 30–39 | 532 (16.4) | 463 (20.9) | 69 (6.7) |  |
| 40–49 | 1234 (38.1) | 887 (40.1) | 347 (33.8) |  |
| 50–60 | 1319 (40.7) | 709 (32.1) | 610 (59.3) |  |
| Education level, n (%) |  |  |  | <0.001 |
| Primary or middle | 969 (29.9) | 546 (24.7) | 423 (41.2) |  |
| High school or college | 1707 (52.7) | 1197 (54.1) | 510 (49.6) |  |
| University and above | 564 (17.4) | 469 (21.2) | 95 (9.2) |  |
| Marital status, n (%) |  |  |  | <0.001 |
| Unmarried | 102 (3.2) | 96 (4.3) | 6 (0.6) |  |
| Married | 3059 (94.4) | 2062 (93.2) | 997 (97.0) |  |
| Other | 79 (2.4) | 54 (2.5) | 25 (2.4) |  |
| Smoking status, n (%) |  |  |  | <0.001 |
| Never/Ever | 1409 (43.5) | 1012 (45.8) | 397 (38.6) |  |
| Current | 1831 (56.5) | 1200 (54.2) | 631 (61.4) |  |
| Drinking status, n (%) |  |  |  | <0.001 |
| Never/Ever | 1890 (58.3) | 1374 (62.1) | 516 (50.2) |  |
| Current | 1350 (41.7) | 838 (37.9) | 512 (49.8) |  |
| DASH score, n (%) |  |  |  | 0.205 |
| <20 | 573 (17.7) | 388 (17.5) | 185 (18.0) |  |
| 20–21 | 1083 (33.4) | 762 (34.5) | 321 (31.2) |  |
| 22–23 | 1034 (31.9) | 703 (31.8) | 331 (32.2) |  |
| ≥24 | 550 (17.0) | 359 (16.2) | 191 (18.6) |  |
| Physical activity, n (%) |  |  |  | 0.744 |
| Low | 35 (1.1) | 26 (1.2) | 9 (0.9) |  |
| Moderate | 226 (7.0) | 154 (7.0) | 72 (7.0) |  |
| High | 2979 (91.9) | 2032 (91.9) | 947 (92.1) |  |
| BMI (kg/m2), n (%) |  |  |  | 0.710 |
| <25 | 1559 (48.1) | 1074 (48.6) | 485 (47.2) |  |
| 25–30 | 1417 (43.7) | 962 (43.5) | 1417 (44.3) |  |
| ≥30 | 264 (8.2) | 172 (8.0) | 264 (8.6) |  |
| Shift work, n (%) |  |  |  | <0.001 |
| Never/Ever | 1292 (39.9) | 944 (42.7) | 348 (33.8) |  |
| Current | 1948 (60.1) | 1268 (57.3) | 680 (66.2) |  |
| Sleep duration (hour), n (%) |  |  |  | 0.034 |
| <6 | 399 (12.3) | 254 (11.5) | 145 (14.1) |  |
| ≥6 | 2841 (87.7) | 1958 (88.5) | 883 (85.9) |  |
| Hypertension, n (%) | 1093 (33.7) | 635 (28.7) | 458 (44.6) | <0.001 |
| Diabetes, n (%) | 465 (14.4) | 252 (11.4) | 213 (20.7) | <0.001 |
| Dyslipidemia, n (%) | 1321 (40.8) | 837 (37.8) | 484 (47.1) | <0.001 |
| Snore, n (%) | 956 (29.5) | 661 (29.9) | 295 (28.7) | 0.491 |
| Sleepdrug, n (%) | 151 (4.7) | 104 (4.7) | 47 (4.6) | 0.871 |

DASH, dietary approaches to stop hypertension; MET, metabolic equivalent of task; BMI, body mass index; HDL-C, high density lipoprotein cholesterol; LDL-C, low density lipoprotein cholesterol.

**Table S2** Additive interactions between shift work and insomnia on odds of carotid plaque. China, 2017

| Additive interaction terms ^a^ | Total |
| --- | --- |
| Relative excess risk due to interaction, RERI (95% CI) | 0.018 (-0.454 to 0.491) |
| Attributable proportion due to interaction, AP (95% CI) | 0.010 (-0.244 to 0.264) |
| Synergy index, S (95% CI) | 1.022 (0.584 to 1.788) |

RERI, relative excess risk due to interaction; AP, attributable proportion due to interaction; CI, confidence intervals;

^a^ If there is no biological interaction, RERI and AP are equal to 0 and S is equal to 1.

Adjusted for age, educational level, BMI (categorical), smoking, drinking, shift work, sleep duration (categorical), sleep drug, snore, hypertension, diabetes and dyslipidemia.


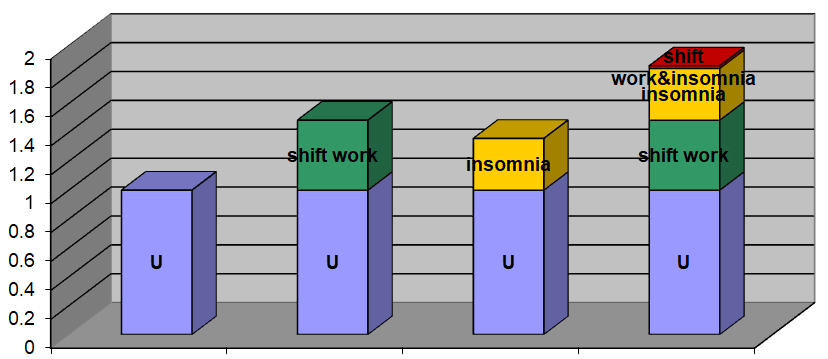


**Figure S1** Relative risk with contributions from different exposure categories marked in additive interaction. U is the common reference category.

**Table S3** The Odds Ratio (OR) of carotid plaque in steelworkers with insomnia compare to non-insomnia. Multivariate logistic regression. China, 2017

| Characteristics | Total, n (%) | OR (95% CI) |
| --- | --- | --- |
| Insomnia |  |  |
| No | 2097 (64.7) | 1.00 |
| Yes | 1143 (35.3) | 1.37 (1.15 to 1.63) |

Adjusted for age, educational level, BMI (categorical), smoking status, drinking status, shift work, sleep duration (categorical), sleep drug, snore, hypertension, diabetes, dyslipidemia, heat stress, noise, dust and carbon monoxide.

**References**

1. **GBZ/T 192.1–2007 Determination of dust in the air of workplace. Part 1: Total dust concentration.** [ <http://niohp.chinacdc.cn/zyysjk/zywsbzml/201210/t20121012_70522.htm>]

2. **GBZ 2.2–2007 Occupational exposure limits for hazardous agents in the workplace. Part 2: Physical**

**agents.** [<http://niohp.chinacdc.cn/zyysjk/zywsbzml/201303/t20130329_79199.htm>]

3. **GBZ/T 189.7–2007 Measurement of physical agents in workplace. Part 7: Heat Stress.** [<http://niohp.chinacdc.cn/zyysjk/zywsbzml/201210/t20121012_70527.htm>]

4. **GBZ/T 229.2–2010 Classification of occupational hazards at workplaces. Part 2: Occupational exposure**

**to chemicals.** [<http://niohp.chinacdc.cn/zyysjk/zywsbzml/201210/t20121012_70489.htm>]

5. **GBZ/T 160.28–2004 Methods for determination of inorganic carbon compounds in the air of workplace.** [<http://niohp.chinacdc.cn/zyysjk/zywsbzml/201210/t20121015_70624.htm>]

6. **GBZ/T 229.4–2012 Classification of occupational hazards at workplaces. Part 4: Occupational exposure**

**to noise.** [<http://niohp.chinacdc.cn/zyysjk/zywsbzml/201307/t20130715_84934.htm>]

7. **GBZ/T 189.8–2007 Measurement of physical agents in workplace. Part 8: Noise.** [<http://niohp.chinacdc.cn/zyysjk/zywsbzml/201210/t20121012_70526.htm>]
